# Nucleic Acid-Based Field-Effect Transistor Biosensors

**DOI:** 10.3390/bios16020095

**Published:** 2026-02-03

**Authors:** Haoyu Fan, Dekai Ye, Xiuli Gao, Yuan Luo, Lihua Wang

**Affiliations:** 1 Institute of Materiobiology, College of Sciences, Shanghai University, Shanghai 200444, China; nana7mi@shu.edu.cn (H.F.); yedk@zjlab.ac.cn (D.Y.); 2 Zhangjiang Laboratory, 100 Haike Road, Shanghai 201210, China; 3 State Key Laboratory of Transducer Technology, Shanghai Institute of Microsystem and Information Technology, Chinese Academy of Sciences, Shanghai 200050, China; 4 Shanghai Collaborative Innovation Center of Intelligent Sensing Chip Technology, Shanghai University, Shanghai 200444, China

**Keywords:** field-effect transistor, biosensor, nucleic acid probe, framework nucleic acid, biomarkers

## Abstract

The demand for rapid and highly sensitive sensing technologies is increasing across diverse fields, including precise disease diagnosis, early-stage screening, and real-time environmental monitoring. Field-effect transistor (FET)-based sensing platforms have shown tremendous potential for detecting target molecules at extremely low concentrations, owing to their ultrahigh sensitivity, label-free and amplification-free operation, and rapid response. In recent years, the rapid advancement of nucleic acid probe design and interfacial engineering has markedly accelerated the development of FET sensors, leading to the emergence of nucleic acid-based FET (NA-FET) biosensors. Beyond their fundamental role in nucleic acid detection, the integration of nucleic acid aptamers and framework nucleic acids has greatly expanded NA-FET biosensors’ applicability to a wide range of analytes and multiplexed detection. At the same time, advances in semiconductor materials have endowed the NA-FET biosensor with highly efficient signal transduction and diverse device architectures, enabling successful proof-of-concept demonstrations for various clinically and environmentally relevant molecular biomarkers. Furthermore, the integration into portable, wearable, and implantable devices has laid a solid foundation for their future development into real-world applications. This review summarizes recent cutting-edge progress in NA-FET biosensors, highlights key design strategies and performance improvements, and discusses current challenges, future development directions, and their prospects for practical applications.

## 1. Introduction

In recent years, driven by the growing demands of precision medicine, early disease screening, and real-time environmental surveillance, biosensor has entered a new phase of technological advancement. The ability to detect diverse classes of biomarkers with ultra-sensitivity, high specificity, rapid turnaround, and in a portable format has become critically important in the development of modern biosensing technologies [1]. Conventional laboratory-based methods, such as enzyme-linked immunosorbent assays (ELISAs) and mass spectrometry, offer excellent analytical accuracy and robustness. However, they typically rely on bulky instrumentation, skilled operators, and time-consuming workflows, which limit their applicability in point-of-care testing (POCT) and on-site, real-time analysis. Consequently, there remains an urgent demand for innovative biosensing modalities that couple ultrahigh sensitivity and rapid signal transduction with simplified operation and straightforward system integration and miniaturization.

**Figure 1:**
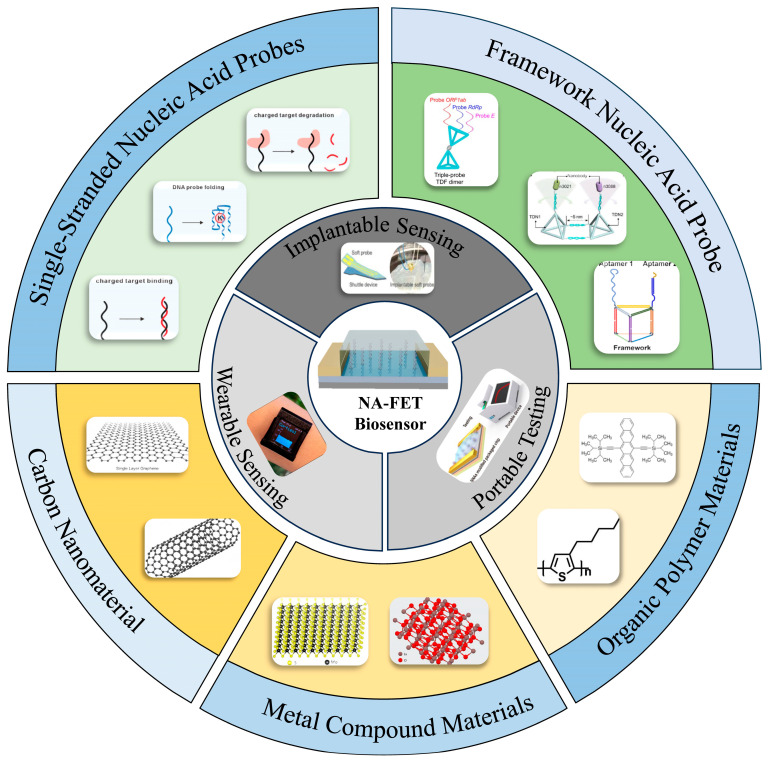
Probe design of nucleic acid field-effect transistors (outer upper green part), types of channel materials (outer lower yellow part), and practical applications (inner circle gray part).

Field-effect transistor (FET) biosensors have emerged as highly promising candidates owing to their unique signal transduction mechanism and favorable semiconductor properties. Since their initial proposal [2], FET-based biosensors have been regarded as one of the most attractive platforms for achieving ultrasensitive and real-time detection, benefiting from their intrinsic signal amplification, fast electrical response, and excellent compatibility with device miniaturization. In particular, novel material-based FETs—exemplified by graphene, carbon nanotubes, and conjugated polymers—have significantly enhanced the sensitivity and response speed of biochemical sensing owing to their exceptionally high carrier mobility, outstanding chemical stability, large specific surface area, and good biocompatibility [3,4,5]. Despite these advances, the overall performance of FET biosensors remains strongly constrained by the precise engineering of the interfacial biorecognition layer [6,7]. Widely used protein-based recognition elements (e.g., antibodies) offer high specificity but suffer from several intrinsic limitations, including susceptibility to denaturation, substantial batch-to-batch variability, and a restricted repertoire of target molecules. These shortcomings directly compromise the detection capabilities of FET biosensors and restrict their broader utility in bioanalytical applications (Figure 1).

The rapid progress in DNA-based molecular probes has opened new avenues for overcoming these limitations in FET biosensing. Nucleic acid aptamers, often referred to as “chemical antibodies,” can be obtained through in vitro selection and exhibit target affinity and specificity comparable to those of antibodies. At the same time, they provide unique advantages, including precise sequences, high chemical and thermal stability, and facile, site-specific chemical modification [8,9]. These features render aptamers attractive as robust and rationally designable recognition units for FET platforms. More importantly, advances in framework nucleic acid now enable the precise construction of three-dimensional, ordered interfacial architectures—such as DNA tetrahedra and DNA origami nanostructures—on FET channel surfaces in a modular, bottom-up manner [10,11]. Such interfacial engineering strategies allow for oriented, homogeneous, and well-controlled immobilization of biorecognition probes, thereby maximizing the accessibility of their active sites. In parallel, they offer fine control over the distance between probe molecules and the semiconductor surface, which is critical for optimizing charge transport and signal transduction. Collectively, these attributes can substantially enhance the sensitivity, reliability, and device-to-device reproducibility of FET biosensors at the molecular level. In addition, the intrinsic addressability of DNA nanostructures enables the co-immobilization of multiple distinct probes on a single FET device, providing a viable route toward high-throughput, multiplexed detection [12].

In this review, we provide a systematic and comprehensive overview of recent advances in nucleic acid-based FET biosensors (NA-FET biosensors). We first outline the fundamental operating principles of FET biosensors and introduce key performance metrics, thereby establishing a basis for the subsequent discussion. We then focus on the diverse DNA elements employed at FET sensing interfaces, ranging from single-stranded aptamers to more complex structured probes (e.g., hairpin motifs and G-quadruplexes). Particular emphasis is placed on framework nucleic acids—especially DNA tetrahedra and DNA origami—as interfacial engineering tools, with detailed discussion of their construction methods, inherent advantages, and the mechanisms by which they improve sensing performance in terms of sensitivity, specificity, and stability. We further highlight representative applications of NA-FET biosensors in several frontier areas, including the detection of biomarkers associated with major diseases, and applications in portable, wearable, and implantable systems. Finally, we discuss the current challenges facing this emerging field and provide a perspective on future development directions, including synergistic integration with artificial intelligence, flexible electronics, and single-molecule detection technologies.

## 2. Basic Structure and Working Principles of NA-FET Biosensor

### 2.1. The Basic Structure of Field-Effect Transistor Biosensors

**Figure 2:**
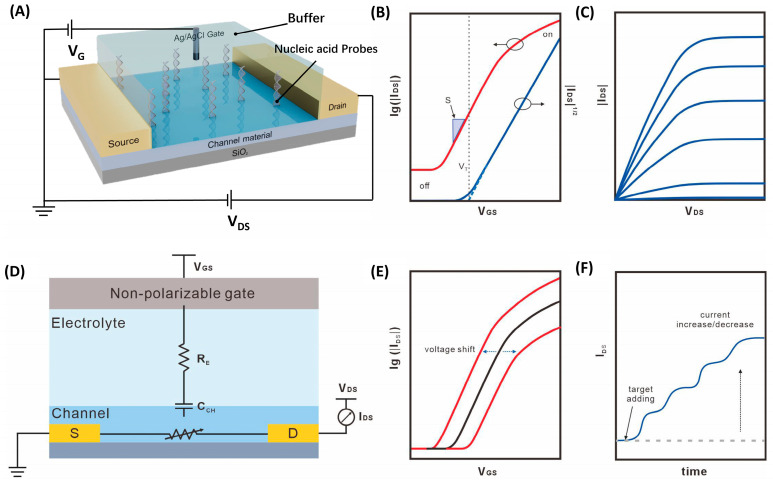
(**A**): The basic structure and external circuit of the nucleic acid FET. (**B**): The transfer characteristic curve and loop of the enhancement-mode-FET. (**C**): The typical output curve of the FET. (**D**): The cross-sectional circuit structure diagram of the FET. (**E**): The displacement of the transfer characteristic curve of the FET. (**F**): The IT curve when measuring the target response.

As shown in Figure 2A,D, a typical FET is a three-terminal device. Its basic structure consists of an electrode layer (source, drain, and gate), a semiconductor channel layer, and an insulating layer (dielectric). Ohmic contacts are formed between the semiconductor channel and the source and drain terminals via metal electrodes. The gate electrode is separated from the semiconductor channel by an insulating layer (e.g., electrolyte in biosensors), forming a parallel-plate capacitor structure, which is the physical basis for electric field control [13,14]. Depending on the gate position relative to the channel, devices can be configured as top-gate or bottom-gate, among other geometries, to suit different integration and sensing requirements [13,15].

FET is a voltage-controlled semiconductor device that modulates the current between its source and drain terminals by using an electric field to control the concentration of charge carriers (electrons or holes) within a semiconductor channel. Unlike conventional bipolar transistors, FETs offer advantages such as high input impedance, low power consumption, and ease of integration [16]. These properties have not only established FETs as core components of modern integrated circuits but have also enabled their development into highly sensitive, real-time biosensing platforms—FET biosensors—by integrating biological recognition events with electrical signal transduction. Their key advantage lies in the ability to directly and label-free convert the binding of target biomolecules (e.g., DNA, proteins, ions) at the sensing interface into an amplifiable electrical signal, providing a powerful tool for point-of-care diagnostics, environmental monitoring, and related fields [17].

### 2.2. Performance Parameters of NA-FET Biosensors

The core of FET biosensor operation is the electrostatic gate-control effect [15,18,19]. The fundamental electrical characteristics of the device are characterized by a set of core physical parameters. The main considerations are the threshold voltage (V_th_), on/off current ratio (I_on_/I_off_), field-effect mobility (μ), and transconductance (g_m_). The threshold voltage (V_th_) is the minimum voltage required for the electric field-induced charge to form a conductive channel in a field-effect transistor. This voltage is determined by the difference in work functions between the metal and the semiconductor [16]. In biosensing, target binding often causes a characteristic shift in V_th_, which serves as a direct detection signal.

The on/off current ratio (I_on_/I_off_) is a parameter used to characterize the switching capability of a field-effect transistor, defined as the ratio of I_Dmax_ to I_Dmin_ under a certain source–drain voltage (V_ds_). I_Dmax_ is typically expressed as the drain current of the FET and is related to gate leakage and channel layer resistivity. Field-effect mobility (μ) is the average drift velocity of carriers under a unit electric field strength. A higher mobility indicates that more carriers pass through the conducting channel within a unit time, reflecting the ability of electrons and holes to migrate within the semiconductor under different electric fields. When the field-effect transistor operates in the linear region, that is, when $\left|V_{ds}\right|\ll \left|V_{gs}-V_{th}\right|$, the current between the source and drain of the n-channel field-effect transistor can be expressed by Formula (1) [20]:

(1) $$\begin{array}{c} I_{ds}=\frac{W}{L}\mu C_{i}V_{ds}\left(V_{gs}-V_{th}\right) \end{array}$$

Here, μ represents the mobility, V_gs_ is the gate voltage, V_ds_ is the source–drain voltage, C_i_ is the capacitance of the dielectric per unit area, and W and L are the channel width and length, respectively [16]. This equation highlights the significance of mobility, saturation velocity, and channel length in field-effect transistors. By using Equation (2) to perform a linear fit on the transfer curve, the slope of the fitted curve is utilized to calculate μ through Equation (2).

(2) $$\mu =\frac{Lg_{m}}{WC_{i}V_{ds}}$$

where *g_m_* is the transconductance, defined by

(3) $$\begin{array}{c} g_{m}\equiv \frac{\partial I_{d}}{\partial V_{gs}}\;V_{ds}=const \end{array}$$

Its physical meaning is that for every 1-volt change in the gate-source voltage, the drain current changes by how many amperes. The larger the transconductance gm, the stronger the control ability of the gate voltage over the drain current, and the more sensitive the device is.

In addition, the purity of semiconductors, the quality of crystals, the size of grains, and the contact of electrodes will also have a significant impact on the mobility. These interrelated parameters collectively form the performance foundation of the FET as a signal transducer and amplifier [13].

Hardware performance evaluation of FETs typically requires the measurement of three characteristic curves (Figure 2B,C,F): output curve (I_ds_–V_ds_), transfer characteristic curve (I_ds_–V), and real-time current curve (I_ds_–T). The output curve is the variation in the drain-source current when the drain voltage is scanned at different V_gs_, and is typically used to calculate the device resistance and the voltage range of current saturation. The transfer characteristic curve is a curve that constantly changes the gate voltage and monitors the current between the source and the drain while the source–drain voltage remains constant. The magnitude of the gate voltage directly and continuously regulates the conductivity of this channel, which in turn determines the level of the drain–source current (I_ds_). This control relationship is fully captured by the transfer characteristic curve (I_ds_ vs. V_gs_) (Figure 2E). This is the core curve that describes the “field-effect” control characteristics and provides key parameters of the FET, such as the threshold voltage (V_th_), the switching ratio (I_on_/I_off_) [21], etc.

### 2.3. Sensing Mechanisms of NA-FET Biosensors

The integration of FETs with DNA probes constitutes a powerful technology in the fields of genetic testing and molecular diagnostics. The working principle of NA-FET biosensors primarily relies on two physical mechanisms: the direct electrostatic gating effect [22,23] and interface potential modulation based on conformational changes [24].

The most direct and widely studied mechanism is the electrostatic gating effect from the negative or positive charge on target molecules. When a charged target is captured by a DNA probe immobilized on the FET channel surface, it effectively introduces additional fixed negative charges into the gate sensing region. This charge change modulates the electric field at the channel surface [14,25]: for an n-type channel, negative charges repel electrons, depleting channel carriers and decreasing conductance; the opposite occurs for a p-type channel. This effect is equivalent to applying an additional bias to the gate, ultimately causing a horizontal shift in the transfer characteristic curve (manifested as a V_th_ shift) or a real-time change in I_ds_ under a fixed bias. This mechanism is straightforward but is sensitive to solution ionic strength and pH, which can screen the charge on target molecules. Furthermore, the detection signal may be weak for target molecules with low intrinsic charge [22,26].

To overcome the dependency on the target’s intrinsic charge and improve detection universality and sensitivity, sensing strategies based on nucleic acid conformational changes have emerged, with aptamer-based sensing being a prominent example [27]. Nucleic acid aptamers are single-stranded DNA/RNA molecules that can fold specifically and bind to targets (e.g., ions, small molecules, and proteins) with high affinity. In this design, the aptamer probe itself is immobilized on the channel surface with a specific initial charge distribution. The presence of the target molecule induces a conformational transition in the aptamer from a random coil to a specific three-dimensional structure (e.g., G-quadruplex [28,29] or stem-loop [29]). This dramatic structural rearrangement can significantly alter the spatial distribution of charge, dipole moment, or the average distance of the probe molecule from the channel surface at the interface, thereby inducing a much larger change in interfacial potential than that caused by the target’s own charge. For instance, the potassium ion-induced formation of a G-quadruplex structure can gather and push the negatively charged phosphate backbone away from the surface, greatly modulating the potential distribution within the electrical double layer [26]. This approach is independent of the target’s charge properties, vastly expanding the range of detectable analytes and often yielding higher signal gain.

In practical device configurations, NA-FET biosensors are typically configured with an electrolyte as a dielectric layer, and the gate voltage is applied using a non-polarizable electrode. The DNA probe is usually functionalized on the surface of the channel material with an electric double-layer (EDL) formation in an electrolyte environment. With the formation of the EDL [30], any minor disturbance of charge or potential at the electrode/electrolyte interface will alter the charge distribution on the surface of the channel material. This will cause a change in the conductivity of the field-effect transistor channel, converting biochemical signals into measurable and observable electrical signals in real time [14,25]. (such as a shift in I_ds_ or threshold voltage V_th_). This structural evolution with low operation voltages grants NA-FET biosensors with unprecedented sensitivity.

Although NA-FET has the potential for high sensitivity, its application in actual physiological environments is hindered by a fundamental physical limitation—the Debye screening effect. Due to the high ionic strength of biological media, biomolecules are surrounded by counterions and form an electric double layer (EDL) on the channel surface. As a result, the effective charges of the target molecules and the sensing interface are severely screened, thereby weakening the electrostatic interaction between them [31]. The calculation of the Debye length is as follows:

(4) $$\lambda_{D}=\frac{1}{\sqrt{4\pi l_{B}\sum_{i}p_{i}z_{i}^{2}}}$$

l_B_ is the Bjerrum length, Σ_i_ is the sum of all ion species, and ρ_i_ and z_i_ are the concentration and valence of ion species i, respectively. Under typical physiological conditions with high salt concentrations, such as 0.15 M NaCl, λ_D_ is usually less than 1 nanometer. The traditional view holds that if the distance between the charge center of the target molecule and the FET channel exceeds this nanoscale Debye length, electronic detection is almost impossible [31,32]. However, most of the molecules to be detected, such as antibodies (10–15 nm) or longer DNA strands (about 10 nm), are much larger than λ_D_. This inherent size mismatch poses a fundamental challenge to charge sensing. Due to the presence of Debye screening, only those charges that are tightly bound within the λ_D_ range and close to the semiconductor surface can effectively couple to the semiconductor channel and produce a gate effect [33].

To address this issue, many methods have been proposed. According to Equation (4), changing the ionic strength of the buffer solution will affect the charge screening effect of the medium, thereby causing a change in the event detection quantity. Therefore, desalinated solutions can be used to increase the Debye length. Sebastian et al. demonstrated that when the buffer concentration was reduced from 1 × PBS to 0.1 × PBS, the sensing response of complementary DNA hybridization increased from 12% to 80% [34]. Other methods include the morphology of curved graphene [35], modification of the graphene surface [36], and the use of shorter receptors to shorten the distance between the target analyte and the channel surface [37].

This expression above all clearly indicates that enhancing NA-FET biosensor performance requires concurrent optimization of the charge/potential change induced by the interfacial biorecognition event (probe chemistry and immobilization strategy) and the efficiency of the device’s own electrical signal transduction (low-dimensional materials, device physics, and structural design).

### 2.4. Performance Evaluation Indicators of NA-FET Biosensors

When an FET is applied to biomolecular detection, its performance must be evaluated using a standardized set of application-oriented metrics, including specificity, sensitivity, LOD, linear range, response time, etc. Sensitivity is a core metric, referring to the ratio of the device’s electrical response (e.g., ΔI_ds_ or ΔV_th_) to the change in analyte concentration, i.e., the slope of the calibration curve. Within a certain concentration range, the analyte concentration and the current value between the source and drain show a strong linear correlation. The S can be calculated by the following formula, where I_d_ is the drain current at the concentration of C, $I_{d_{0}}$ is the background drain current. This concentration range is the linear range.

(5) $$\begin{array}{c} S=\frac{I_{d}-I_{d_{0}}}{C} \end{array}$$

The LOD is the lowest concentration of analyte that can be reliably detected with a given confidence level (typically corresponding to a signal-to-noise ratio of 3). According to the definition of IUPAC (International Union of Pure and Applied Chemistry), the detection limit is the minimum amount or lowest concentration of the substance to be tested that can be detected from a sample within a given confidence level (usually 95% or 99%). The calculation formula is as follows:

(6) $$\begin{array}{c} LOD=\frac{k\cdot S_{b}}{S} \end{array}$$

Here $S_{b}$ is the standard deviation of the blank control experiment, and S is the calibration curve, that is, the sensitivity calculated by Formula (4). k: A factor (e.g., 3 or 3.3) for confidence. It reflects a combined effect of the device’s sensitivity and noise level. Specificity examines the sensor’s ability to accurately identify the target molecule while excluding other interfering substances in a complex sample matrix, which primarily depends on the biorecognition properties of the interfacial probes (e.g., DNA). Additionally, response time (time to reach 90% of the steady-state response) reflects detection kinetics speed [17], while the dynamic range and linear range define the concentration interval over which the sensor operates effectively.

In practical applications, factors such as reproducibility, long-term stability, integrability, and production cost are also critical determinants for the technology’s path toward practical utility and commercialization. Therefore, optimizing NA-FET biosensors is a systematic engineering endeavor requiring synergistic advances across multiple disciplines, including materials science, device physics, interface chemistry, and biotechnology.

## 3. Nucleic Acid Probe

In NA-FET biosensors, the nucleic acid probes immobilized on the channel surface serve as the “molecular recognition arms” that confer high selectivity to the device. Their specific binding to target molecules (e.g., DNA, RNA, proteins, and ions) triggers changes in the interfacial physicochemical properties, which are subsequently transduced into measurable electrical signals by the highly sensitive FET channel. Consequently, the design of nucleic acid probes and the strategies used for interfacial assembly are central determinants of overall sensor performance. From the perspective of structural and functional evolution, nucleic acid probes can be broadly categorized into two main classes: conventional single-stranded nucleic acid probes and framework nucleic acid nanoprobes with well-defined three-dimensional architectures. The synergistic integration of these probes with diverse channel materials has collectively driven the rapid advancement of NA-FET technology.

### 3.1. Single-Stranded Nucleic Acid Probes

**Figure 3:**
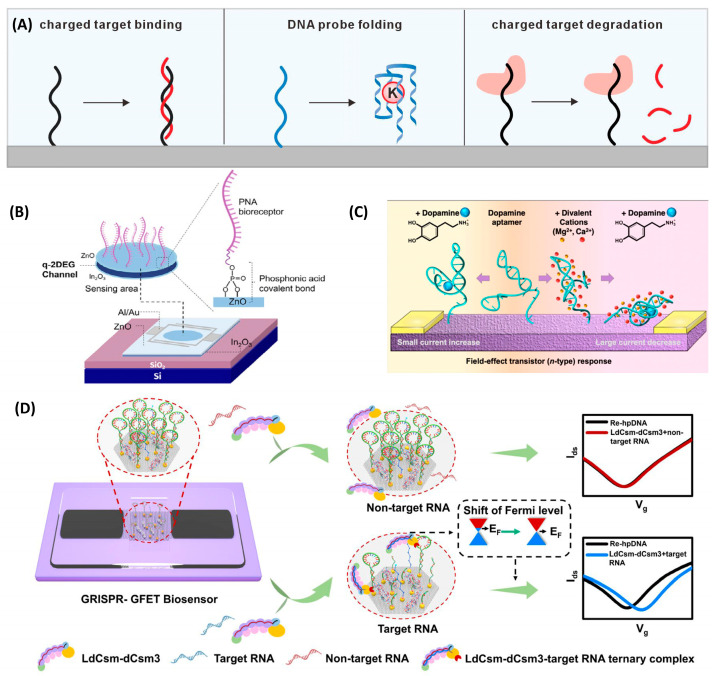
(**A**) Schematic diagram of the principle of the interface regulation mechanism of three types of nucleic acid probes. (**B**) Custom-synthesized peptide nucleic acid (PNA) probes were covalently immobilized on the ZnO channel surface via phosphonic acid groups for the specific capture of target miRNA. The PNA probes used have an electrically neutral pseudo-peptide backbone. Adapted with permission from ref. [40], which is an open-access article distributed under a Creative Commons Attribution 4.0 International License. (**C**) The way metal ions (Mg^2+^ and Ca^2+^) act as allosteric regulators in aptamer-based field-effect transistors helps aptamers better capture dopamine. Adapted with permission from ref. [41]. Copyright 2021 American Chemical Society. (**D**) The LdCsm-dCsm3 complex activated by the target RNA continuously cleaves the re-hpDNA at the solid-liquid interface, causing it to dissociate from the graphene surface and a sudden drop in the charge density, which leads to a significant positive shift in the Dirac point. Adapted with permission from ref. [29], which is an open-access article distributed under a Creative Commons Attribution 4.0 International License.

Single-stranded nucleic acid probes represent the foundational building blocks of NA-FET sensors, exhibiting robust detection ability to diverse molecular targets. The most straightforward sensing modality is based on complementary strand hybridization. An ssDNA probe is immobilized on the channel surface via, for example, π–π stacking interactions or Au–S bonds. Upon hybridization with complementary target DNA or RNA in solution, the additional negative charges introduced by the phosphate backbone of the duplex modulate the carrier density in the channel (Figure 3A). Since Shin and colleagues first employed ssDNA self-assembled monolayers on Au-gated MOSFETs for DNA detection in 2004 [38], this strategy has been widely adopted. For instance, Gao et al. utilized partial fragments of single-stranded DNA complementary to the targeted miRNA, which were modified on flexible graphene field-effect transistors, achieving a detection limit as low as 10 fM for the target miRNA [39]. However, such direct electrostatic sensing is highly susceptible to the intrinsic background charge of the probe.

To overcome these limitations, peptide nucleic acids (PNAs) have been introduced as an excellent alternative probe. The PNA replaces the negatively charged sugar–phosphate backbone with a charge-neutral N-(2-aminoethyl)glycine backbone. This structural modification brings several critical advantages: (i) it eliminates interference from the intrinsic background charge of the probe, and (ii) the neutral backbone experiences reduced electrostatic repulsion toward negatively charged DNA targets, resulting in stronger binding affinity at low ionic strength and improved discrimination between fully complementary sequences and single-base mismatches [42,43,44]. In FET-based sensing, PNA–DNA hybridization can induce more pronounced positive shifts in threshold voltage and a reduction in saturation current compared to conventional DNA–DNA duplex formation. PNA can solve the problem of low sensitivity of traditional NA-FET due to the shielding of charges by counterions, and it can also distinguish complementary DNA from single-base mismatched DNA and non-complementary DNA [45,46,47]. For instance (Figure 3B), Ghamdi modified the In_2_O_3_/ZnO heterojunction interface with PNA probes featuring an electrically neutral pseudo-peptide backbone, replacing traditional DNA probes. This approach offers higher specificity, binding affinity, and resistance to enzymatic degradation, along with an extremely low detection limit and the ability to distinguish single-nucleotide mismatches with ultra-high specificity [40].

The advent of nucleic acid aptamers has dramatically expanded the sensing capabilities of NA-FET platforms. Aptamers selected via SELEX can recognize a broad spectrum of targets—including metal ions [24,26,48], small molecules [49], proteins [36], and even whole viruses [50]—with antibody-like specificity and affinity [27]. Their key sensing advantage lies in the reversible and pronounced conformational rearrangements that occur upon target binding (e.g., transitions from a flexible single-stranded state to compact stem–loop or G-quadruplex structures). Such structural transitions modulate the spatial distribution of charge, the dipole moment, and the average distance between the probe and the transistor channel, thereby producing a strong gating effect that does not necessarily rely on the intrinsic charge of the target. For example, a DNA aptamer responsive to Cu^2+^ appears as numerous uniformly distributed small white spherical features under AFM in its single-stranded state. Upon specific recognition of Cu^2+^, it folds into a double-stranded structure, appearing as flocculent bright features in AFM images, and the surface roughness of the FET channel increases from 2.86 nm to 4.80 nm. Using this aptamer, a low detection limit of 10 nM for copper ions can be achieved [51]. Moreover, Nako et al. reported an allosteric aptamer that requires the cooperative binding of Mg^2+^/Ca^2+^ and dopamine, thereby enabling complex logic-gated sensing behaviors (Figure 3C) [41].

Among the various target-induced conformational changes in DNA aptamers, the G-quadruplex is particularly common and has been extensively investigated (Figure 3A). This secondary structure, prevalent in both DNA and RNA, is formed by guanine (G)-rich sequences and is highly polymorphic. G-quadruplexes can adopt a variety of topologies, depending on the orientation of the G-strands (parallel or antiparallel), the configuration of the connecting loops (lateral, diagonal, or double-lateral loops), and the molecular stoichiometry [52]. Consequently, there have been numerous reports exploiting G-quadruplex formation in FET-based sensors [24,53]. For instance, Zhuang et al. [28] employed the IGA3 aptamer, which exists predominantly in a loose, unfolded conformation in insulin-free buffer. In the presence of insulin, the aptamer binds via a specific recognition site, and this binding event acts as a molecular trigger that induces a drastic conformational change into a stable and compact G-quadruplex. The aptamer thereby folds from an extended strand into a three-dimensional architecture. As a result, the negatively charged insulin and the DNA phosphate backbone are brought within the Debye length of the graphene surface, leading to n-type doping of the graphene channel. Under a fixed gate voltage, the channel current decreases monotonically during the binding process, and a detection limit of 35 pM in 1 × PBS buffer is achieved.

In addition, charge doping of the channel surface can be modulated not only by the binding of target molecules to immobilized nucleic acids, but also inversely by the cleavage of these nucleic acids. CRISPR–Cas-based collateral cleavage mechanisms have introduced a new paradigm for NA-FET sensing (Figure 3A). For example, Chen et al. immobilized RNA reporter strands on an IGZO-FET surface; upon activation by target RNA, Cas13a nonspecifically cleaves the reporters, causing a rapid decrease in interfacial charge density and enabling ultrasensitive RNA detection down to the single-copy-per-microliter level in simulated throat swab samples, suggesting a pathway to achieve rapid point-of-care detection and scalable epidemic screening [54]. Sun et al. employed a similar strategy (Figure 3D), using Cas10 to cleave hairpin DNA immobilized on Au nanoparticles assembled on graphene; the resulting shifts in the Dirac point enabled amplification-free quantification of target RNA [29].

### 3.2. Framework Nucleic Acid Nanoprobes

Despite their versatility, ssDNA probes suffer from random orientation and poorly controlled spatial distribution at the interface, which limits the uniformity and optimization of sensing performance. DNA nanotechnology leverages the programmability of Watson–Crick base pairing to self-assemble DNA into structurally precise, mechanically robust framework nucleic acids—such as DNA tetrahedra, triangular prisms, origami structures, and spherical nucleic acids—thereby providing revolutionary tools for engineering next-generation high-performance sensing interfaces [11,55,56].

**Figure 4:**
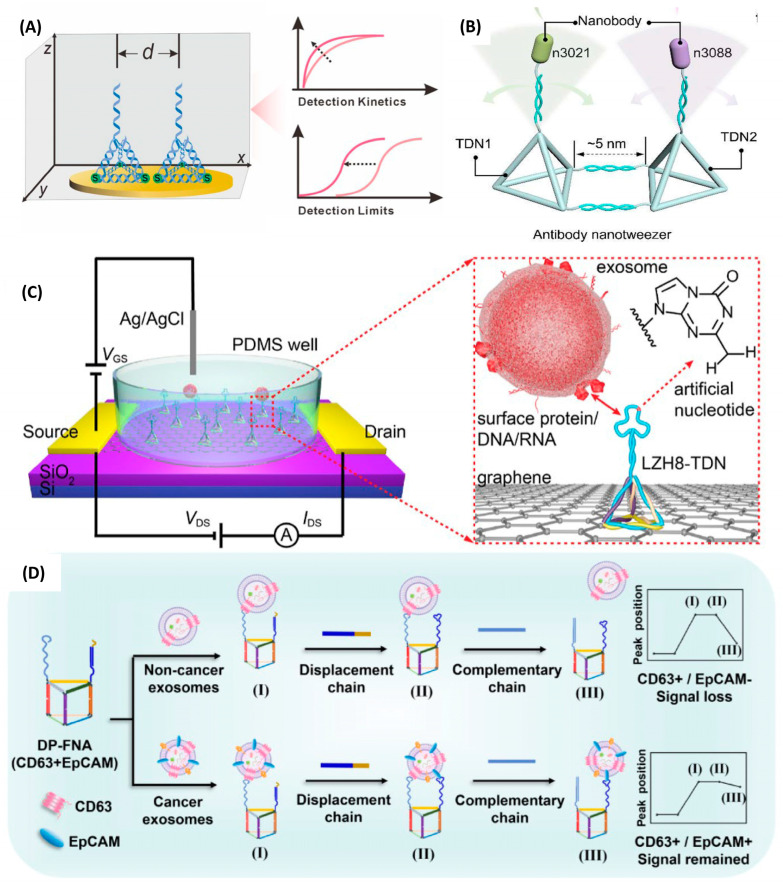
(**A**) Schematic diagram of the enhancement of framework nucleic acids. (**B**) Two DNA tetrahedra act as rigid scaffolds, carrying bivalent nanobodies to form antibody nanotweezers with spatial adaptability. Adapted with permission from ref. [61]. Copyright 2024 American Chemical Society. (**C**) Using tetrahedral DNA nanostructures (TDNs) as rigid scaffolds, aptamers were uprightly immobilized on the surface of graphene for the detection of proteins on the surface of exosomes. Adapted with permission from ref. [62]. Copyright 2022 American Chemical Society. (**D**) Two aptamers targeting different proteins, CD63 and EpCAM, were modified on the DNA triangular prism. Through a programmed “capture-unlock-release” process, logical analysis of the two proteins on the EV surface was conducted, and the signal was retained to specifically recognize the exosomes of cancer cells. Adapted with permission from ref. [63]. Copyright 2025 American Chemical Society.

DNA tetrahedra (TDNs) are three-dimensional nucleic acid frameworks characterized by simple design, facile synthesis, high structural stability, and pronounced rigidity [57]. Since their inception several decades ago, a substantial body of work has reported the use of DNA tetrahedra in biosensing, with widespread applications in nucleic acid detection, small-molecule sensing, and related areas. TDNs are most commonly integrated with electrochemical techniques for target detection [58,59,60], but in recent years, the rapid development of field-effect transistors (FETs) has provided a superior bioanalytical platform that opens an entirely new avenue for exploiting TDNs. In the available literature, the majority of framework nucleic acids immobilized on FET channel surfaces are DNA tetrahedra, which have matured into a well-established system(Figure 4A).

The excellent modularity at the tetrahedral vertices allows the attachment of customized probes, such as single-stranded DNA (ssDNA) targeting specific gene sequences. Ma et al. [64] integrated an ssDNA probe complementary to the target circulating tumor DNA (ctDNA) into one vertex strand of the TDN, achieving a detection limit as low as 50 fM for a 21 bp model sequence. The same platform was also effective for longer targets (90 bp and 120 bp), which more closely resemble authentic ctDNA, and even produced stronger signals. Wu et al. incorporated a native DNA aptafimer recognizing the Alzheimer’s disease biomarker Aβ-42 into one vertex of a TDN and achieved a detection limit down to 5 aM for Aβ-42 with a response time of less than 5 min in full serum, far outperforming devices employing ssDNA aptamers alone [65]. In another study (Figure 4C), a tetrahedral-structured graphene FET, functionalized with an artificial nucleic acid aptamer, enabled highly sensitive detection of hepatocellular carcinoma-derived exosomes with a low detection limit of 242 particles/mL and could distinguish positive serum samples within 9 min [62].

Moreover, attaching biomolecules such as proteins or cholesterol to the vertices or edges of TDNs can endow specific capture capabilities even in the absence of a dedicated DNA aptamer. In 2021, Dai et al. functionalized a MoS_2_-FET biosensor with DNA tetrahedra bearing biotin at the vertex distal to the interface [66]. Streptavidin was subsequently linked to this biotin, and an anti-PSA antibody was further conjugated via biotin–streptavidin chemistry, yielding a complex TDN–biotin–streptavidin–biotin–anti-PSA architecture capable of selectively capturing PSA. This sensor achieved a detection limit of 1 fg/mL with a linear dynamic range from 1 fg/mL to 100 ng/mL in undiluted healthy human serum. Wu et al. [67] linked two DNA tetrahedra in a tip-to-tip fashion, with one tetrahedron anchored to the graphene surface of an FET and the other suspended in the electrolyte. Three different probes were attached to the three basal vertices of the upper tetrahedron; their cooperative binding enhanced overall affinity, enabling amplification-free detection of SARS-CoV-2 RNA in artificial saliva at concentrations of 0.025–0.05 copies/μL. To further address limitations in binding affinity, Wang et al. proposed an “antibody nano tweezer” (Figure 4B) design in which two nanobodies are precisely arranged by a DNA tetrahedron scaffold, achieving a detection limit of 0.5 aM, approaching the theoretical single-molecule level [61].

Biosensing based on solid-state electronics has long faced a persistent challenge when monitoring trace analytes in solution, namely, insufficient analyte recognition at the sensing interface. Electrostatic preconcentration using graphene FETs has emerged as a promising solution. In 2021, Wei and colleagues [68] functionalized liquid-gated FETs with rigid DNA tetrahedra bearing flexible arms and utilized the electric field to preconcentrate low-abundance analytes, thereby enabling direct detection of SARS-CoV-2 nucleic acids. This platform was capable of accurately identifying COVID-19–positive samples in 10-in-1 pooled nucleic acid tests. One year later, the same group [69] developed a molecular electromechanical system comprising an aptamer probe conjugated to the tip of a flexible ssDNA cantilever, which is tethered to a DNA tetrahedron. The tetrahedron is anchored on the graphene FET via three amino groups at its base. Under electrostatic actuation, the cantilever bearing the bound target bends toward the semiconductor interface, reducing the distance between the analyte and the sensing surface. This configuration enables ultrasensitive and highly specific detection of proteins, small molecules, ions, and nucleic acids in biological fluids, while also exhibiting antifouling properties. The study demonstrated detection of SARS-CoV-2 nucleic acids in clinical samples within 0.1–4 min, with a detection limit down to 0.3 aM. Collectively, these works highlight a new paradigm in which DNA tetrahedra on FETs synergistically integrate device hardware with the physical properties of molecular probes.

The packing density and size of DNA tetrahedra immobilized on FETs also critically influence the performance of nucleic acid–FET sensors. One report indicated that, when graphene is used as the channel material, a TDN edge length of 17 bp combined with a 5-base spacer in the probe overhang produces a larger Dirac-point shift, enabling more efficient signal transduction while avoiding entanglement of the overhang [25]. In addition, Ma et al. found that a spacer of four thymine residues between the tetrahedral vertex and the probe yields a superior biosensor response compared with spacers of two or six thymines [64].

The common and mature structure of the DNA tetrahedron can also bring problems, such as insufficient binding sites and weak binding capacity [7,13,66]. To meet more complex sensing demands, additional innovative framework structures are continually being developed. Spherical nucleic acids [70], consisting of Au nanoparticle cores densely modified with thiolated DNA, form three-dimensional probe shells with high loading capacity and can be readily deposited on various FET surfaces. They have been employed for ultrarapid detection of targets such as DNA and ATP. Min et al. were the first to introduce a DNA triangular prism into carbon-nanotube FETs [71]; the three edges of the prism can be independently functionalized, allowing simultaneous targeting of three genes of respiratory syncytial virus RNA and enabling RSVRNA’s diluted clinical samples detection down to 0.1 copies μL^−1^ within 40 s (Figure 4D). Ai et al. exploited the UV sensitivity of DNA origami structures by immobilizing them on graphene FETs; UV-induced degradation of the origami altered the interfacial π–π interactions, achieving an ultra-high sensitivity of 0.005 kJ per square meter, which is more than five times that of existing UV meters [72].

## 4. Channel Materials

The channel material serves as the physical platform that transduces microscopic biorecognition events into macroscopic, measurable electrical signals. Its electronic properties (e.g., carrier mobility and bandgap) and surface chemistry directly dictate the baseline noise, signal gain, stability, and ultimate limit of detection of the sensor. Ideally, a channel material should combine high charge carrier mobility, excellent biocompatibility, and an interface that supports stable and well-controlled biofunctionalization. With the advancement of materials technology, the channel materials used in FETs have shown a clear trend of evolving from conventional bulk materials toward low-dimensional and functionally engineered systems. These materials have an extremely high surface-to-volume ratio, and their conductive channels are entirely located on the surface or near the surface [73,74]. Representative examples include silicon nanowires [75], indium oxide (In_2_O_3_) [76,77], transition-metal carbides, metal sulfide [78], or carbonitrides (MXenes) [79,80], as well as the recently emerged black phosphorus [81]. In the following sections, several important representative material systems will be introduced.

### 4.1. Carbon Nanomaterials

#### 4.1.1. Graphene

**Figure 5:**
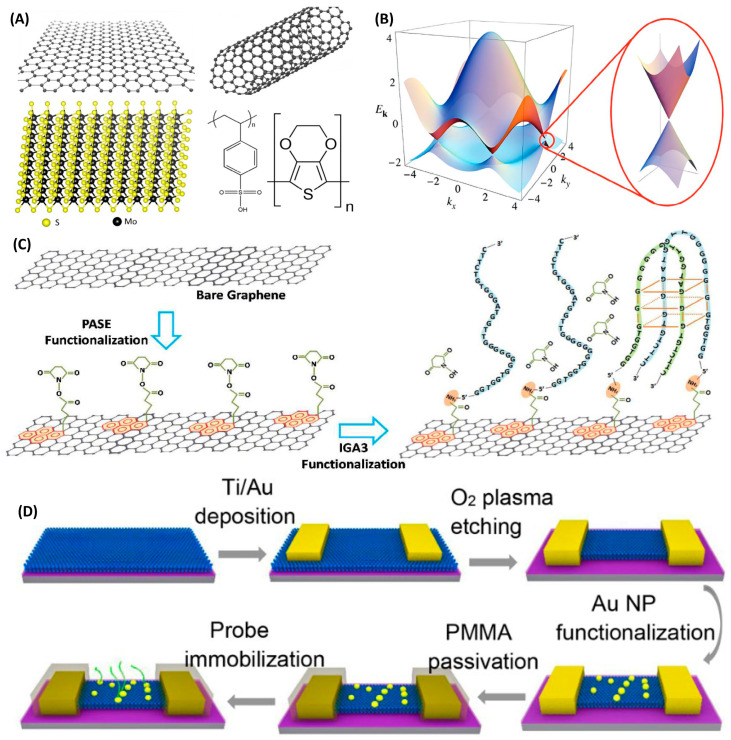
(**A**): Schematic diagrams of the structures of graphene, carbon nanotubes, monolayer molybdenum disulfide, and molecular formulas of organic semiconductors. (**B**): Schematic diagram of the energy level structure of single-layer graphene and the Dirac cone. Adapted with permission from ref. [83]. Copyright 2009 American Physical Society. (**C**): The modification steps of nucleic acids on graphene. First, the PASE is fixed by π–π interaction, and then the nucleic acid chain is linked by covalent condensation of the nucleic acid with an amino group and the N-hydroxy succinimide ester (NHS ester). Adapted with permission from ref. [28]. Copyright 2017 American Chemical Society. (**D**): Steps for modifying nucleotide chains on molybdenum disulfide FETs. Adapted with permission from ref. [96]. Copyright 2019 American Chemical Society.

As the first isolated two-dimensional material, graphene has become one of the most widely used platforms in NA-FETs owing to its atomic thickness, ultrahigh carrier mobility, and large specific surface area (Figure 5A). Graphene can be regarded as one of many allotropes of carbon [82]. Its two-dimensional lattice is composed of sp^2^-hybridized carbon atoms arranged in a honeycomb lattice, with carbon atoms forming hexagons. Three valence electrons form strong in-plane covalent bonds, while the fourth electron remains in the p orbital to form an out-of-plane π bond. Electrons in the p orbitals can easily detach between atoms, resulting in graphene’s unique linear dispersion relation [83]. The honeycomb structure gives the Brillouin zone of graphene central symmetry, and there is a zero bandgap point between the valence band and the conduction band, also known as the Dirac point [17,22]. Its linear energy and zero bandgap dispersion enable flexible tuning of the Fermi level via gate voltage or surface adsorbates (Figure 5B). This is intuitively reflected in shifts in the Dirac point in transfer characteristics, providing a highly sensitive and easily readable electrical parameter for biosensing. Achieving efficient and stable immobilization of nucleic acid probes on graphene is critical for constructing high-performance sensors.

The most prevalent strategy relies on the bifunctional linker 1-pyrenebutyric acid N-succinimidyl ester (PASE). The pyrene moiety noncovalently adsorbs onto the sp^2^-hybridized carbon lattice of graphene through strong π–π interactions, causing minimal disturbance to graphene’s intrinsic electronic properties (Figure 5C). The NHS ester group at the other end readily undergoes covalent condensation with amine (-NH_2_)-modified nucleic acid probes, forming robust amide bonds [25,84,85]. This immobilization approach has been extensively validated as both reliable and efficient.

Graphene FET (G-FET) biosensors constructed using PASE have enabled high-performance detection of a wide variety of targets, covering various molecules such as DNA [86], miRNA [87], proteins [61,88], and neurotransmitters [89]. For the regulation of the surface properties of graphene, Hwang et al. developed a nucleic acid FET biosensor featuring a deformable, wrinkled graphene channel [35]. Peptide nucleic acid (PNA) probes were immobilized on the graphene surface, and a clever, low-cost “heat-shrink” treatment was employed to induce nanoscale wrinkles in the graphene. In this architecture, salt ions in the solution are partially excluded from the concave regions of the wrinkles, effectively increasing the local Debye length and thereby enhancing the performance of the FET device. By anchoring specific aptamers, G-FETs have also achieved ultrasensitive recognition of proteins, small molecules, and even intact viral particles, underscoring the potential of graphene as a universal sensing platform.

#### 4.1.2. Carbon Nanotubes

Single-walled carbon nanotubes (SWCNTs) are composed of sp^2^-hybridized carbon atoms and can be regarded as seamless hollow cylinders formed by rolling up a single graphene sheet (Figure 5A), with diameters typically ranging from 0.48 to 2.0 nm. The chiral vector, usually denoted by a pair of integers (n, m) [90], defines the rolling direction and thereby determines both the tube diameter and its electronic properties. Depending on their chirality, SWCNTs can exhibit metallic behavior with no bandgap and excellent electrical conductivity [91], or semiconducting characteristics with a finite bandgap that can be modulated by a gate voltage.

Given the nanosized diameter of SWCNTs, any charge carried by a nucleic acid molecule adsorbed on their surface can directly modulate the carrier concentration along the entire conductive channel. This renders SWCNT-based FETs extremely sensitive to electrostatic perturbations induced by biomolecules, theoretically enabling single-molecule detection. Benefiting from their atomic-scale thickness, exceptionally high carrier mobility (up to ~10^5^ cm^2^·V^−1^·s^−1^), and outstanding surface charge sensitivity, SWCNTs represent ideal channel materials for constructing next-generation, highly sensitive, label-free point-of-care diagnostic devices. Functionalization strategies for SWCNTs can be broadly divided into two categories. The first employs aromatic molecules [92] (e.g., 1-pyrenebutyric acid) that noncovalently adsorb via π–π stacking. This approach preserves the near-perfect lattice structure and outstanding electronic properties of the nanotubes. The second strategy introduces ultrathin dielectric layers (e.g., Al_2_O_3_) and Au nanoparticles (AuNPs) as intermediates, followed by covalent immobilization of thiolated nucleic acids through Au–S chemistry [93,94]. This method provides reliable, uniform, and universal functionalized surfaces, resolves the stability issue, and is highly suitable for wafer-level manufacturing.

Leveraging these strategies, CNT-FETs have demonstrated outstanding sensing performance. They enable ultrasensitive detection of biotin [95], DNA biomarkers [64] (such as circulating tumor DNA of triple-negative breast cancer), and cellular metabolic activities [70], with limits of detection (LODs) in some cases reaching the femtomolar (fM) or even attomolar (aM) range [91].

### 4.2. Metallic Compound

Carbon nanomaterials represented by graphene have demonstrated outstanding charge mobility in the field of nucleic acid detection, bringing extremely high detection sensitivity. However, their zero bandgap characteristic limits the on–off ratio (I_on_/I_off_) of the device [16,82], thereby affecting the signal-to-noise ratio of the sensor. Metal compound semiconductors of sulfides and oxides have well-defined bandgaps. This enables the device to be completely “turned off”, achieving an extremely high on–off ratio in nucleic acid detection, which means a cleaner background signal and a greater current change. Moreover, the maturity of the processing technology of metal compounds gives the sensor an advantage in large-scale production and uniformity [97].

The metal compound materials applied to NA-FET are mostly one-dimensional, two-dimensional, and quasi-two-dimensional, and can generate an ultra-sensitive response to external stimuli [17]. Layered transition metal dichalcogenides (TMDs) are composed of layered crystal structures, in which chalcogen atoms and metal atoms are bonded by covalent bonds to form layered stacking arrangements (Figure 5A). Adjacent layers are connected by van der Waals forces [98]. Molybdenum disulfide is a typical layered metal dichalcogenide, featuring 2H and 3R layered structures. Its layered structure is stacked in a hexagonal symmetrical manner, which is more stable than the 3R structure. It also has atomically flat surfaces and can be easily prepared by exfoliation. Compared with graphene, molybdenum disulfide naturally has a larger energy band gap, making MoS_2_ a true semiconductor material that can effectively control electron flow. The existence of the band gap also enables molybdenum disulfide field-effect transistors to be completely turned off, thus having a high on–off ratio. Monolayer MoS_2_ possesses a direct bandgap of approximately 1.8 eV, enabling FETs with very high on/off current ratios (often exceeding 10^8^) and extremely low off-state currents. This substantially reduces static power consumption and improves signal-to-background ratios, giving MoS_2_-based devices unique advantages for biosensing [16,99].

A common strategy for immobilizing nucleic acids on MoS_2_ exploits its spontaneous redox reaction with chloroauric acid to in situ grow uniformly distributed AuNPs on the surface, which then serve as anchors for thiolated probes through strong Au–S bonds [100] (Figure 5D). Using this method, Hossain et al. constructed a microfluidic sensing interface on a MoS_2_ field-effect transistor with a high-density deposition of gold nanoparticles (AuNPs) [101]. By immobilizing single-stranded or double-stranded DNA aptamers as recognition elements, label-free detection of bisphenol A (BPA) with a detection limit as low as 1 pg mL^−1^ was achieved in 1 × PBS buffer. This immobilization method was more robust than that on a graphene interface and could be reused after a simple PBS buffer rinse. In addition, similar to graphene, MoS_2_ can also be noncovalently modified with PASE via van der Waals interactions, providing further flexibility for its biofunctionalization [102,103].

Other metal oxides, such as indium gallium zinc oxide (IGZO), zinc oxide (ZnO), indium oxide (In_2_O_3_), and indium tin oxide (ITO), are also widely used in field-effect transistor sensors [20,104]. It is also usually processed into low-dimensional nanomaterials such as nanowires and nanofilms to enhance the sensing performance [104].

ZnO nanomaterials are typical representatives of oxide semiconductors. Shariati et al. [105] took advantage of the extremely high surface-to-volume ratio of ZnO nanowires and their ease of doping with Mo to develop ZnO nanowire nucleic acid field-effect transistors for the detection of hepatitis B virus. The Mo dopant introduced vacancy sites in the ZnO lattice, which facilitated the adsorption of DNA and charge transfer. Another study adopted aluminum-doped ZnO films to make the ZnO films more compact. By using the positive charges on the ZnO surface to fix DNA aptamers, it was made more suitable as the channel material for high-performance and long-life field-effect transistor biosensors [106].

IGZO is a new type of semiconductor material that has emerged in recent years. It can be processed on a large scale at relatively low temperatures and can be easily functionalized on the surface through traditional organosilane chemistry and cross-linking reactions, forming a stable biochemical interface that facilitates the immobilization of biological probes. It also features low-voltage operation and a high on/off ratio [97]. Hwang et al. [107] used oxygen plasma to generate hydroxyl groups on the IGZO surface and then covalently attached single-stranded probe DNA via APTES to prepare an IGZO nucleic acid FET with recyclable capabilities, achieving specific capture of SARS-CoV-2 DNA. The large-scale fabrication capability of IGZO reduces the cost of the sensor and enhances its practicality. The following year, the same team used the same approach to design a sensor for detecting MMP-9 protein, a biomarker for dry eye disease, achieving sub-microliter sample detection [108].

Among metal oxide nanomaterials, In_2_O_3_ has been extensively reported in the field of NA-FETs [109,110]. It exhibits excellent electronic properties, is easy to fabricate into ultrathin near two-dimensional structures, and has an extremely high surface area-to-volume ratio [77,111]. Compared to IGZO and ZnO mentioned above, In_2_O_3_ demonstrates long-term stability in electrolyte solutions (similar to the physiological environment in the body). Additionally, ultrathin (2–3 nm) semiconductor films of In_2_O_3_ prepared by the sol-gel chemical method in solution have high uniformity, ensuring high statistical confidence when calibrating biomarker concentrations. These factors are crucial for implantable biosensors [112]. Therefore, it has been widely applied in implantable and wearable devices and implantable devices. For instance, Zhao et al. developed In_2_O_3_-based flexible implantable probe FETs [76,113] and skin patch NA-FETs for detecting cortisol [114]. The nucleic acid functionalization of In_2_O_3_ is typically achieved by treating the In_2_O_3_ surface with APTES and glutaraldehyde to capture amino-modified nucleic acid probes [110] or by treating the surface with MalC10PA as a bifunctional linker to capture thiol-modified nucleic acid probes [77,109].

### 4.3. Organic Polymer Materials

Organic semiconductors (OSCs) constitute a broad class of π-conjugated small molecules and polymers whose electronic structures can be finely tuned through molecular design and chemical synthesis [115,116]. By modifying the backbone and side chains, their energy levels, charge-transport characteristics, solubility, and interfacial properties can be independently optimized. Owing to their solution processability and compatibility with low-temperature, large-area fabrication on flexible substrates, OSCs are particularly attractive for biointegrated electronics and wearable sensing platforms.

Organic field-effect transistors (OFETs) typically operate in accumulation mode, where charge transport occurs in a two-dimensional channel at the interface between the OSC and the dielectric layer. Consequently, the interfacial properties directly determine the charge carrier mobility (μ) and threshold voltage (V_th_). When biomolecules interact with the OSC or the dielectric interface, they can alter the interfacial charge density, dipole orientation, or semiconductor morphology, thereby modulating the source–drain current (I_ds_).

Compared with inorganic FETs, OFETs offer several advantages, including low cost, printability, mechanical flexibility (suitable for wearable and patch-type sensors), good biocompatibility, and the absence of a requirement for a reference electrode [117,118]. Massey et al. used TIPS-pentacene (6,13-bis(triisopropylsilylethynyl) pentacene) as the organic semiconductor channel material and designed a multilayer ultrathin polymer dielectric stack (PMMA/PDMS/PVA) to physically isolate the gate and the organic semiconductor from the electrolyte, developing a laboratory prototype of a soft fluidic integrated organic electrolyte-gated field-effect transistor [119]. Its modified aptamer probe can specifically bind to αSyn monomers, enabling the detection of Parkinson’s disease. Up until now, OFET-based biosensors have been extensively reported for the detection of proteins and nucleic acids [119,120,121].

The choice of channel material and the associated interfacial engineering are central to optimizing NA-FET performance. Integrating the intrinsic properties of these materials with carefully engineered nucleic acid probes—particularly framework nucleic acids—and implementing optimized immobilization strategies to construct stable, homogeneous, and efficient biointerfaces is key to driving NA-FET technology from laboratory demonstrations toward real-world applications.

## 5. Clinical Diagnosis and Point-of-Care Applications

NA-FET biosensors, by virtue of their ultrahigh sensitivity, low power consumption, and compatibility with standard microfabrication processes, are reshaping the landscape of biomolecular detection. By integrating highly specific nucleic acid probes, the detectable targets of NA-FET biosensor have expanded far beyond nucleic acid sequences to encompass proteins, exosomes, small molecules, ions, and even whole pathogens. This provides a powerful technological platform for applications ranging from highly sensitive molecular diagnostics to real-time, dynamic health monitoring. Table 1 summarizes the sensing capabilities of NA-FET biosensors for different types of health-related biomarkers.

### 5.1. Biomarker Detection in Disease Diagnosis

Accurate quantification and real-time analysis of biomarkers are fundamental to early disease screening, precise diagnosis, prognosis evaluation, and therapeutic monitoring [127]. NA-FET biosensor enables label-free, real-time, and ultrasensitive detection of a wide array of key biomarkers, effectively overcoming the limitations of conventional approaches such as ELISA and PCR, which often require complex workflows, bulky instrumentation, and labeling or amplification steps [128].

#### 5.1.1. Nucleic Acid Detection

As carriers of genetic information, nucleic acids (DNA/RNA) are central molecular markers of cancer, genetic disorders, and infectious diseases through sequence variations, methylation patterns, and dysregulated expression. NA-FET biosensors are particularly well-suited for rapid analysis of trace nucleic acid biomarkers such as circulating tumor DNA (ctDNA), microRNAs (miRNAs), and single-nucleotide polymorphisms (SNPs).

Due to its crucial role in regulating gene expression and its association with pathological conditions, miRNA has become a highly promising biomarker for liquid biopsy. Compared with traditional methods that rely on DNA amplification and require expensive and bulky optical detection equipment, the field-effect transistor detection scheme is mature and well-developed. In recent years, there have been numerous reports on the detection of miRNA-141, miRNA-21, miRNA-155, and other types using different kinds of field-effect transistors [40,64,87,94,102,129]. These detection schemes provide excellent references for the diagnosis of cancer diseases such as prostate cancer, breast cancer, cervical cancer, liver cancer, and colon cancer.

**Figure 6:**
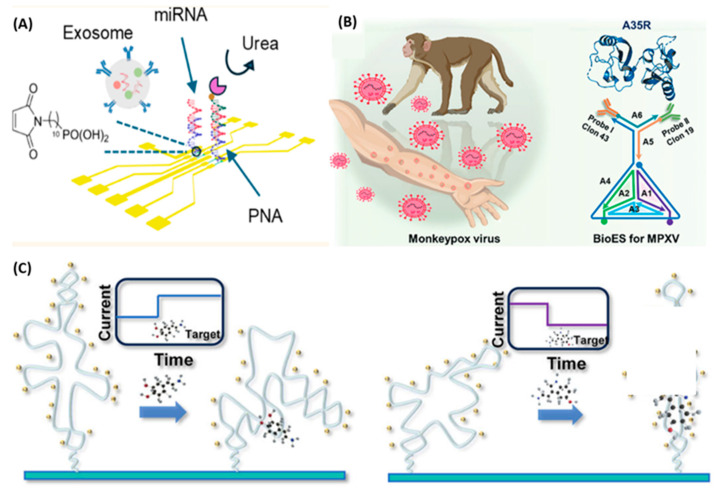
(**A**) In_2_O_3_ nanoribbon field-effect transistor biosensors modified with PNA for ultrasensitive detection of circulating miRNA in exosomes. Adapted with permission from ref. [77]. Copyright 2025 American Chemical Society. (**B**) Using DNA tetrahedra as the probe base, monkeypox antibodies are linked on Y-shaped scaffolds to specifically detect monkeypox antigen proteins. Adapted with permission from ref. [130] Copyright 2019 Wiley-VCH Verlag GmbH. (**C**) Nucleic acid aptamers that can be regulated by neurotransmitter small molecules on the surface of channel materials to change spatial conformation. Adapted with permission from ref. [131]. which is an open-access article distributed under a Creative Commons Attribution 4.0 International License.

For example, Zhao et al. [77] employed an In_2_O_3_ nanoribbon FET to construct a PNA–target RNA–secondary DNA–biotin sandwich architecture (Figure 6A). In this design, electrically neutral PNA probes improved the signal-to-noise ratio for binding negatively charged target miRNAs, achieving a remarkable label-free limit of detection (LOD) of 0.72 aM and enabling clear discrimination of miRNAs with only 2–3 base mismatches, demonstrating excellent selectivity. Wejdan and colleagues functionalized In_2_O_3_/ZnO heterojunction thin-film transistors with custom-designed PNA probes and monitored threshold voltage shifts induced by captured miR-141, achieving an LOD of 0.6 fM and providing a new tool for prostate cancer diagnosis [40].

Single-nucleotide polymorphisms (SNPs) refer to variations in a single nucleotide within the genome [40]. SNPs account for over 50% of human disease-causing mutations, making discriminating single-base differences critical for the diagnosis of genetic diseases. Balderston et al. [86] innovatively combined the CRISPR–Cas system with graphene FETs to develop a highly specific detection platform. The principle is that when the guide RNA (gRNA) perfectly matches the target DNA sequence in a sample, the CRISPR–Cas complex tightly binds and unwinds the DNA, generating a pronounced “gating” effect at the graphene surface and producing a measurable electrical signal change; in the presence of mismatches, binding is significantly weaker. Using this strategy, the authors successfully detected pathogenic mutations associated with sickle cell disease and amyotrophic lateral sclerosis (ALS), showcasing outstanding capability for discriminating SNPs.

#### 5.1.2. Protein Detection

Proteins are the direct executors of biological function, and changes in their abundance, post-translational modifications, and conformations are important indicators of disease. NA-FET biosensor can achieve highly specific, label-free protein detection by employing aptamers or antibody–nucleic acid conjugates as recognition elements.

Exosomes are nanoscale vesicles that carry membrane proteins and nucleic acids reflective of their parent cells, and are emerging as highly promising biomarkers for cancer diagnosis [132]. Targeting specific membrane proteins on hepatocellular carcinoma (*HepG2*)-derived exosomes, Chen et al. developed a graphene FET sensor based on artificial nucleic acid aptamers and DNA tetrahedra [62]. In this work, DNA tetrahedra were used to anchor aptamer probes in an upright, well-ordered configuration on the graphene surface, preventing the entanglement issues common with linear probes and substantially enhancing target binding efficiency. The sensor achieved an LOD of 242 particles mL^−1^ for liver cancer–derived exosomes—nearly four orders of magnitude lower than that of conventional electrochemical methods—and was able to differentiate serum samples from liver cancer patients and healthy individuals within 9 min, and it shows almost no response to the exosomes of MIHA (normal liver cells) and PANC (pancreatic cancer cells). Ren et al. also developed a multi-body biomarker capture system based on carbon-nanotube field-effect transistors. This system utilized self-assembled tetrahedral DNA nanostructures (TDN) with Y-shaped DNA frames attached at the top and modified with antibodies against the monkeypox virus *A35R* (Figure 6B), achieving an ultrahigh sensitivity detection limit at the aM level [130].

Focusing on the transmembrane protein CD9 associated with pancreatic cancer-derived exosomes, An et al. immobilized a selected CD9-specific aptamer on MXene (Ti_3_C_2_T_x_) nanosheet channels [133]. The resulting device achieved an ultralow LOD of 641 particles mL^−1^ in complex human serum matrices. Notably, the fabricated electrodes retained stable detection signals (error < 5%) after 6 days of dry storage, demonstrating excellent practical stability.

#### 5.1.3. Small-Molecule Detection

Neurotransmitter imbalance is closely linked to a range of neurological and psychiatric disorders [134]. Zhao et al. functionalized ultrathin In_2_O_3_ semiconductor films with a DNA aptamer specific for serotonin (5-HT), achieving femtomolar-level detectionin inbrain tissue homogenates with second-scale response times [113]. This strategy effectively mitigated interference from electroactive species commonly encountered in traditional electrochemical assays. In addition, Xiao et al. linked four aptamers that undergo conformational changes upon binding events with gold nanoparticles on carbon-nanotube thin-film field-effect transistors (Figure 6C) [131]. The spatial conformational changes altered the charge distribution near the carbon-nanotube channel, achieving femtomolar detection limits for dopamine, serotonin, histamine, and glutamate, as well as more than ten cycles of reuse. The binding of each neurotransmitter induced a characteristic conformational change in its aptamer, modulating the local charge distribution near the CNT channel. This enabled simultaneous detection of four key neurotransmitters with femtomolar LODs in 0.1 × PBS buffer, and the devices showed good reusability (>10 measurement cycles).

Cortisol is a steroid hormone/small molecule biomolecule, which has a strong correlation with mental disorders such as Cushing’s syndrome, major depressive disorder, anxiety disorders, and PTSD [135]. Wang et al. modified flexible In_2_O_3_ FETs with cortisol aptamers and exploited binding-induced conformational changes to modulate channel charge transport, achieving an LOD of 1 pM in artificial sweat—fully covering and even extending below the physiological concentration range of cortisol in sweat [114]. Pursuing ultimate sensitivity, Park and colleagues adopted a different strategy: they fabricated nanopores on MoS_2_ FET surfaces to expose highly active edge sites and immobilized cortisol aptamers at these locations. Upon cortisol binding, negative charges accumulated at the nanopore edges, strongly repelling electrons in the n-type MoS_2_ channel and driving the LOD down to an astonishing 2.76 aM both in human serum and artificial saliva [136].

### 5.2. Point-of-Care and In Situ Applications

The miniaturization, low power consumption, and direct electrical readout of NA-FET biosensors make them highly amenable to integration with microfluidics, readout electronics, and wireless communication modules. This facilitates the transition of testing from centralized laboratories to point-of-care diagnostics, continuous personal health monitoring, and even in vivo, in situ biochemical analysis.

#### 5.2.1. Portable Testing

By integrating NA-FET biosensor with miniaturized readout circuits and disposable microfluidic chips, stand-alone point-of-care testing (POCT) devices or handheld analyzers can be developed. Such systems are in high demand for applications including infectious disease control and on-site food safety screening.

**Figure 7:**
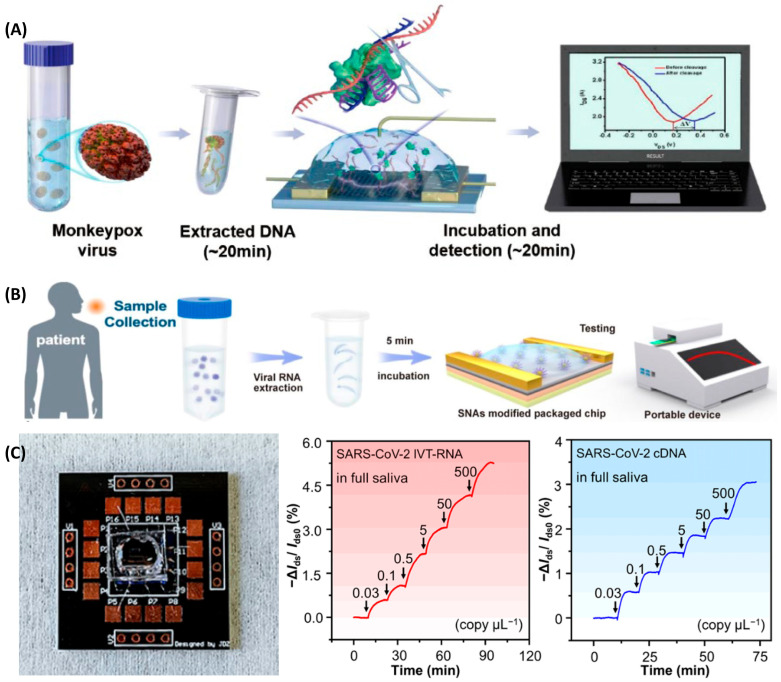
(**A**) Rapid detection of the monkeypox virus, adapted with permission from ref. [137], which is an open-access article distributed under a Creative Commons Attribution 4.0 International License. (**B**) Spherical nucleic acid carbon-nanotube field-effect transistors for rapid detection of enteroviruses, adapted with permission from ref. [70], Copyright 2024 American Chemical Society. (**C**) The artificial in vitro transcribed RNA of *SARS-CoV-2* and the reverse transcription complementary DNA of the RNA virus itself, both detected by the Y-shaped nucleic acid structure, show obvious signal changes within a short period of time. Adapted with permission from ref. [122]. Copyright 2021 American Chemical Society.

Wang et al. [137] developed a GFET sensor with single-stranded DNA (ssDNA) modified on the graphene surface as the reporting probe and combined with the CRISPR/Cas12b system (Figure 7A). It can recognize the target DNA of the monkeypox virus (such as the *B6R* or *F3L* gene), trigger the “trans-cleavage” activity of Cas12b, non-specifically degrade the ssDNA probe fixed on the graphene surface, change the charge density of the sensing interface and the conductivity o f graphene, achieving rapid and ultrasensitive detection without pre-amplification under the laboratory buffer system, with a detection limit as low as 1 aM and only requiring about 20 min. Additionally, the high specificity of the CRISPR/Cas12b system to distinguish different virus strains is of great significance for epidemiological monitoring. Zhang’s team used spherical nucleic acids with gold nanoparticles at the core as probes for CNT-FETs, enabling the detection of enterovirus 71 and respiratory syncytial virus (*RSV*) at the attomolar level on highly integrated chips and portable devices within 100 s in 0.01 × PBS buffer (Figure 7B), notably, this study covered the carbon-nanotube channel with a layer of Y_2_O_3_ insulating layer. This structure isolates the sensitive CNT channel from the complex liquid environment, preventing direct erosion or non-specific interference from the liquid environment, thereby significantly enhancing stability and anti-drift capability. The researchers placed the device in a 0.01 × PBS solution for a 24 h test, and the average change in sensor current was only 2.31%. At the same time, the authors randomly selected 20 FET devices for testing, and the results showed that they had highly uniform transfer characteristic curves with very little variation. It has good application value in clinical applications [70].

During the COVID-19 pandemic, rapid and accurate detection of the virus has become an important demand in responding to the epidemic [67]. The rapid testing potential of the NA-FET biosensor was further highlighted. Wang and his colleagues further integrated DNA tetrahedron-modified g-FETs into portable devices, enabling detection within one minute [138]. Kong et al. developed a graphene FET for the *ORF1ab* and N genes of *SARS-CoV-2*, eliminating the need for any nucleic acid pre-amplification and reducing the detection time to approximately 40 s, which greatly meets the demands of high-throughput on-site screening (Figure 7C) [122]. To address detection escape caused by viral mutations, Zhang et al. [139] used a hafnium fluoride–graphene–hafnium fluoride–gold gate electrode and developed a phenomenological capacitive network model. This approach enables detection of base mismatches and allows real-time, in situ, and time-efficient monitoring of viral biomarkers, somatic mutations, and gene-editing off-target effects.

#### 5.2.2. Wearable Sensing

As personalized medicine shifts from “offline sampling” to “real-time dynamic monitoring” and from “centralized testing” to “personal health management,” the development of continuous monitoring platforms that can be stably integrated with biological interfaces has become a major research focus [140]. However, current wearable and implantable sensors face serious challenges in complex physiological environments [140,141], including signal degradation, poor resistance to biofouling, and constraints on device miniaturization. Nucleic acid field-effect transistors offer a promising solution to these issues and have already shown significant potential in applications such as sweat metabolite analysis, interstitial fluid ion monitoring, and in vivo neurotransmitter tracking.

**Figure 8:**
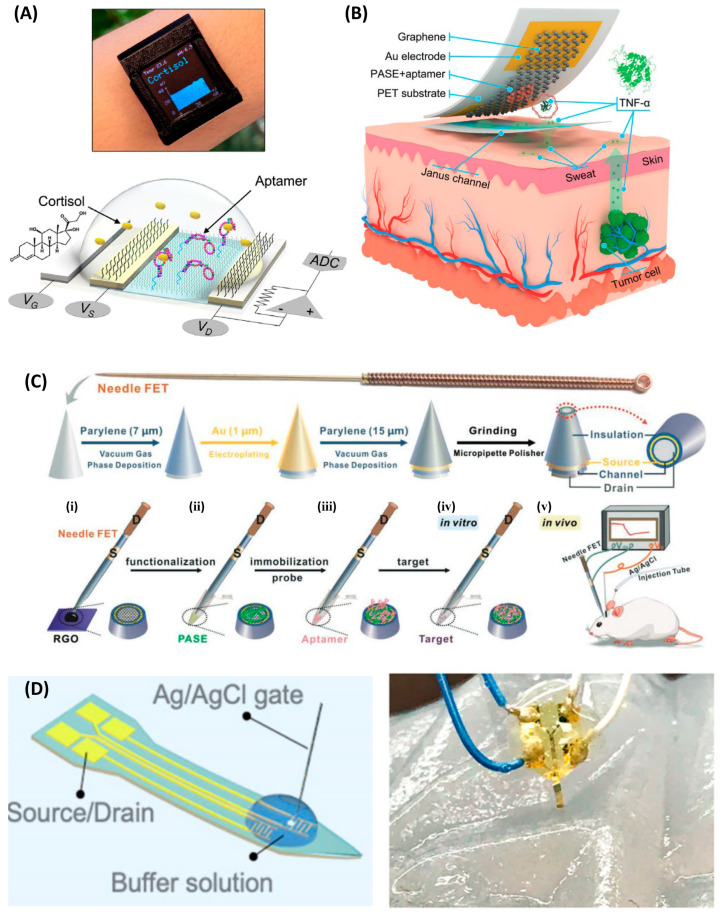
(**A**) Wearable non-invasive cortisol detection system, adapted with permission from ref. [114], which is an open-access article distributed under a Creative Commons Attribution 4.0 International License. (**B**) Schematic diagram of the Janus membrane structure, which uses flexible nucleic acid graphene FET. Adapted with permission from ref. [123], Copyright 2024 Wiley-VCH Verlag GmbH. (**C**) A type of FET acupuncture needle, with the needle body as the drain and the outer metal layer as the source, is used to detect small biological molecules in the mouse brain in real time. Adapted with permission from ref. [89], Copyright 2022 Wiley-VCH Verlag GmbH. (**D**) Silicon-based flexible needle-shaped FET probes can be inserted into the biological brain to detect neurotransmitters in real time with low trauma. Adapted with permission from ref. [76], Copyright 2022 American Chemical Society.

Advances in flexible electronics allow NA-FET biosensors to be fabricated on thin, flexible substrates such as polyimide (PI) and polyethylene terephthalate (PET) [125,142], enabling skin-conformal epidermal electronics or smart patches for noninvasive, continuous monitoring of biomarkers in sweat or interstitial fluid. Sweat contains a wealth of physiological information, but its low analyte concentrations and impurities pose significant challenges. For instance, Wang et al. developed a flexible FET array based on nano-film indium oxide (In_2_O_3_) for cortisol detection [114]. The device was fabricated on a polyimide substrate and used an aptamer deformation to regulate the channel charge distribution. It also incorporated a tape-like thin-film microfluidic channel for adhering to the skin, collecting, and guiding sweat (Figure 8A). Iontophoresis was used to induce sweating, and the data was transmitted to a mobile phone app via Bluetooth. The detection capability achieved a detection limit as low as 1 pM and a response time within seconds in artificial sweat. Furthermore, after 100 bending cycles, the transfer characteristics of the flexible In_2_O_3_ FET remained consistent, with minimal changes in mobility, demonstrating excellent properties in wearable applications.

In view of the extremely low concentration of cytokines in sweat and the presence of a large amount of insoluble impurities, the Huang team developed a Janus membrane with an automatic water collection and filtration function (Figure 8B) [123]. By utilizing gradient surface energy, it can automatically collect sweat and transport it to the sensor surface without external force, while filtering out impurities such as oils and skin scales. Based on a 1 μm PET flexible substrate, aptamer-modified graphene field-effect transistors were prepared, which can highly specifically and sensitively detect the inflammatory cytokine *TNF-α* (LOD 0.31 pM) in the presence of interfering substances such as insulin and amino acids. The long-term stability of the device was verified in the text. The current-voltage (I-V) characteristic curves of the sensor remained stable within 2 to 15 days, demonstrating excellent long-term storage and usage stability. Additionally, the device also exhibited outstanding mechanical stability. After undergoing 50 extreme deformations, the change rates of its transconductance and Dirac point were maintained at relatively low levels.

#### 5.2.3. Implantable Sensing

An implantable NA-FET biosensor can be deployed deep within tissues (e.g., brain and blood vessels) to achieve real-time, in vivo monitoring of biochemical signals, offering transformative tools for neuroscience, chronic disease management, and postoperative care. Key challenges include minimizing invasiveness and ensuring long-term biocompatibility and signal stability.

Zhao et al. developed an implantable NA-FET biosensor for real-time monitoring of serotonin release in the brains of living mice [113]. The device used silicon as the substrate, indium oxide (In_2_O_3_) thin films as the FET channel material, and surface-immobilized DNA aptamers with high specificity for serotonin recognition. The probe adopted a shank-type architecture with the FET sensor integrated at the tip. However, the high stiffness of the silicon shank can trigger immune-mediated inflammatory responses during long-term implantation. One strategy to address this issue is structural optimization to obtain a more compliant and integrated needle-like design. For example, Zhou et al. reported a novel FET configuration based on medical acupuncture needles, featuring a coaxial layered structure in which the needle core serves as the drain and a gold film deposited on an insulating layer functions as the source [89]. With a diameter of only 0.25 mm, this needle is minimally invasive and reduces damage to living tissue.

Biological compatibility can also be improved by changing the substrate material. Using polyimide as the substrate reduces the bending stiffness of the probe to 1.2 × 10^−11^ N·m^2^, several orders of magnitude lower than that of silicon-based probes, thereby greatly enhancing mechanical compatibility with brain tissue and reducing immune responses and glial scarring [76]. Along these lines, Ding et al. developed a nucleic acid-aptamer-gated “water-gated” artificial synaptic organic electrochemical transistor, in which flexible transistor probes implanted into the tail vein of mice enabled real-time monitoring of inflammatory signals in a living sepsis model [124]. The channel material, PEDOT:PSS, is an organic semiconductor with excellent ion–electron conduction and biocompatibility, making it suitable for direct operation in the blood. However, the lack of rigidity complicates implantation, and in their study, an indwelling needle was required to assist insertion into the mouse tail vein. To overcome this limitation, Zhao et al. proposed an assisted implantation strategy for flexible FET on a polyimide substrate: a detachable silicon “shuttle” (Si carrier), exploiting capillary forces, was used to deliver the probe [76]. After implantation, buffer diffusion dissipates the capillary forces, allowing the flexible probe to separate spontaneously from the carrier.

Due to minor differences in manufacturing processes, the baseline current of each transistor (FET) may vary, which can cause significant deviations in practical applications. To address the variability between devices and batches in the aforementioned applications, a calibration response [113,143] is typically employed to minimize the differences among devices. Although there may still be variations in threshold voltage or current baseline between physical devices, this relative change calibration algorithm enables different probes to be comparable when detecting the same concentration of substances. Through the calibration method proposed by Fumiaki N. Ishikawa et al. [144], the coefficient of variation in the response between devices can be reduced from a maximum of 59% to a minimum of 16–19%.

## 6. Conclusions and Outlook

As the demand for highly sensitive, rapid, and portable detection continues to grow, NA-FET biosensors have become a key bridge between cutting-edge bioelectronics and health monitoring. By exploiting the intrinsic sensitivity of FETs to surface charge modulation and incorporating functional DNA elements—such as nucleic acid aptamers, structured DNA probes, and DNA frameworks—into the gate-controlled interface, a wide range of biorecognition events can be directly transduced into amplified changes in source–drain current or threshold voltage. Compared with traditional electrochemical aptamer sensors and optical biosensors, the detection limit of NA-FET can reach the attomole level, which is difficult for electrochemical detection methods to achieve. Compared with optical methods, NA-FET does not require complex sample pretreatment, labeling, and other steps, nor does it need sophisticated instruments. It has a real advantage in the detection of trace biological small molecules. The synergistic design of different channel materials together with sophisticated interface engineering strategies constitutes the core technological foundation for optimizing NA-FET biosensor performance.

Current studies indicate that, on the one hand, high-mobility low-dimensional channel materials provide the physical basis for low-noise, high-gain electrical readout. On the other hand, three-dimensional DNA nanostructures such as DNA tetrahedra and DNA origami can precisely regulate the spatial configuration and charge distribution of probes at the interface, enhancing both specific recognition and signal transduction efficiency. Meanwhile, device platforms such as organic field effect transistors (OFETs) integrated with nucleic acid probes have demonstrated excellent in vivo signal recording capabilities in applications including neurotransmitter detection and inflammation monitoring, thereby laying an important foundation for the development of a flexible implantable NA-FET biosensor.

Despite these advances, NA-FET biosensors still face multiple hurdles on the path toward large-scale practical deployment and clinical translation. First, achieving high selectivity and low false-positive rates in complex biological matrices remains challenging. How to enhance anti-interference performance through antifouling interface layers, multivalent recognition architectures, and ratiometric signal readout strategies is a key question for future research. Second, the electrochemical stability, biocompatibility, and batch-to-batch consistency of devices under long-term operating conditions require further improvement. The absence of unified standardized testing and evaluation protocols also hampers horizontal comparison across studies and poses barriers to clinical regulatory approval. In addition, balancing high performance with low cost, large-area manufacturability, and disposable use will be critical for NA-FETs to progress toward commercialization.

Looking ahead, the development of the NA-FET biosensor is expected to follow a trend of multidimensional, cross-disciplinary integration. First, deep integration with microfluidic chips, portable readout circuits, and wireless communication modules will further drive the NA-FET biosensor from centralized laboratories toward point-of-care testing (POCT) and home self-testing, enabling a transition from single-endpoint measurements to continuous online monitoring. Second, advances in flexible electronics and implantable device technologies will allow an NA-FET biosensor based on organic semiconductors and OFET to better match the mechanical properties of soft tissues, opening up opportunities for long-term in situ monitoring of neural activity, metabolic status, and inflammatory processes. Third, leveraging machine learning and artificial intelligence algorithms to extract features and recognize patterns from complex, multiparametric electrical signals is expected to enable multi-marker panel analysis, individualized threshold setting, and adaptive calibration, thereby significantly enhancing the diagnostic reliability and decision-making value of NA-FETs in real-world settings.

Overall, NA-FET biosensors are gradually evolving from “proof-of-concept” laboratory devices into comprehensive technology platforms that are “engineerable, scalable, and clinically translatable.” With the coordinated advancement of high-performance channel materials, programmable DNA nanostructures, advanced micro/nanofabrication techniques, and intelligent data analytics, NA-FET biosensors are poised to play an increasingly important role in early screening and companion diagnostics for major diseases, environmental and food safety monitoring, personalized health management, and smart wearable/implantable medical devices, becoming a key enabling technology for the deep integration of precision medicine and digital healthcare.

## Figures and Tables

**Table 1:** Summary of the detection performance of NA-FET for different targets.

Target Type	Target	Probe Type	Channel Material	Modification Methods	LOD	Response Time	Refs.
Nucleic Acid	RSVRNA	Framework nucleic acid with ssDNA probes	CNT	-SH	0.1 copies/μL	~40 s	[71]
	miRNA-141	PNA	In_2_O_3_/ZnO	Phosphonic acid anchor groups	0.6 fM	2 h	[40]
	miRNA-21	ssDNA	CNT	Au-S	0.87 aM	-	[94]
	miRNA-21	Framework nucleic acid with ssDNA probes	Graphene	PASE	5.67 × 10^−19^ M	1 h	[87]
	SARS-CoV-2	CRISPR-Cas system	IGZO	SMCC	1 cp μL^−1^	~20 min	[54]
	*E. coli* gene	CRISPR-Cas system	Graphene-MXene	APTES	-	1 h	[80]
	SARS-CoV-2	Framework nucleic acid	Graphene	PASE	0.03 copy/μL	~40 s	[122]
	miR-200b	PNA	In_2_O_3_	(10-BRomodecyl)phosphonic acid	0.72 aM	5 min	[77]
Protein	miRNA-155	CRISPR-Cas system	Graphene	Au-S	427 aM	45 min	[29]
	SOD1 gene	CRISPR-Cas system	Graphene	PBA	6.3 fM	40 min	[86]
	TNF-α	Aptamer	Graphene	PASE	0.31 pM	-	[123]
	CD63, EpCAM, MUC1	Framework nucleic acid with ssDNA probes	Au	Au-S	5.35 × 10^7^ particles/mL	4.5 h	[63]
	SARS-CoV-2 spike protein	Framework nucleic acid with nanobodies	Graphene	PASE	0.5 aM	~50 s	[61]
	Interleukin-6 (IL-6)	Aptamer	PEDOT:PSS	Au-S	0.5 pM	A few minutes	[124]
	HepG2 exosomes Protein	framework nucleic acid with aptamer	Graphene	PASE	242 particles/mL	9 min	[62]
Small biomolecules	PSA	Framework nucleic acid	MoS_2_	Au-S	1 fg/mL	~2 min	[66]
	Dopamine	Aptamer	Graphene	PASE	370 pM	Immediate	[89]
	BPA	Aptamer/dsDNA	MoS_2_	Au-S	1 pg/mL	4 s	[101]
	Dopamine and Serotonin	Aptamer	In_2_O_3_	MBS	1 nM to 1 μM	-	[41]
	Progesterone	Aptamer	PEDOT:PSS.	Au-S	0.5 fM	~5 min	[125]
	ATP	Spherical Nucleic Acids	CNT	Au-O	0.55 ag mL^−1^	100 s	[70]
Metal ion	Hg^2+^	ssDNA	Graphene	Au-S	16 pM	15 min	[26]
	Pb^2+^	dsDNA	Graphene	Au-S	1.9 nM	-	[126]

## Data Availability

No new data were created or analyzed in this study.

## Abbreviations

The following abbreviations are used in this manuscript:

FET	Field-Effect Transistor
DNA	Deoxyribonucleic Acid
GFET	Graphene Field-Effect Transistor
RNA	Ribonucleic Acid
CNT	Carbon Nanotube
LOD	Limit of Detection
NA-FET	Nucleic Acid-Based Field-Effect Transistor
EDL	Electrical Double-Layer
PASE	1-pyrenebutyric acid N-succinimidyl ester
PNAs	Peptide Nucleic Acids
SWCNTs	Single-Walled Carbon Nanotubes
PMMA	Polymethyl methacrylate
PDMS	Polydimethylsiloxane
PVA	Polyvinyl Alcohol
SMCC	N-SucciniMidyl 4-(N-MaleiMidoMethyl)cyclohexane-1-carboxylate
MBS	3-MaleimidobenzoicacidN-hydroxysuccinimideester
PSA	Prostate-Specific Antigen
